# Supplementary material for: Exposure to psychotropic medications and mortality in schizophrenia: a 5-year national cohort study
Source: Psychol Med. 2022 Sep 22;53(12):5528–37. doi: 10.1017/S0033291722002732 (PMC10482725; doi:10.1017/S0033291722002732)
Supplement: Supplementary file 1 [file S0033291722002732sup.zip › S0033291722002732sup002.docx]

**Word count:** abstract: 250; full text: 4000; 2 tables, 3 figures, 50 references, 1 supplementary figure.

**Exposure to Psychotropic Medications and Mortality in Schizophrenia: A 5-Year National Cohort Study**

Ji-Yu Lin^1^, Ling-Ling Yeh^2^, Yi-Ju Pan*^,1,3^

^1^Department of Psychiatry, Far Eastern Memorial Hospital, New Taipei City, Taiwan

^2^Graduate School of Humanities and Social Sciences, Dharma Drum Institute of Liberal Arts, New Taipei City, Taiwan

^3^Institute of Public Health, National Yang Ming Chiao Tung University School of Medicine, Taipei, Taiwan

Running title: Psychotropic medications and mortality in schizophrenia

*Correspondence to Yi-Ju Pan, MD.,Ph.D

Address: Department of Psychiatry, Far Eastern Memorial Hospital, No.21, Sec. 2, Nanya S. Rd., Banciao Dist., New Taipei City, Taiwan

Phone: +886-2-77281264

Fax: +886-2-89660906

Email: [panyiju0211@gmail.com](mailto:panyiju0211@gmail.com); yjpan@mail.femh.org.tw

**Financial support:**

This study was supported by grants from the Ministry of Science and Technology

(MOST 104-2314-B-418-003; MOST 106-2314-B-418-002; MOST 107-2314-B-418-014; MOST 109-2314-B-418 -010; MOST 110-2314-B-418 -004) and Far Eastern Memorial Hospital, Taiwan (FEMH-2016-D-015; FEMH-2017-C-017; FEMH-2018-C-022; FEMH-2019-C-012; FEMH-2020-C-020; FEMH-2021-C-024). The funding body played no role in the study design, analysis, or interpretation of data in this paper.

**Abstract**

**Background.** Relatively few studies have explored the differential contributions of the accumulative dosage of psychotropic medications on mortality in patients with schizophrenia.

**Methods.** We aimed to explore the effects of the exposure dosage of psychotropic medications on mortality during a follow-up period of 5 years with a national cohort of individuals with schizophrenia in 2010. Causes of death were linked through Taiwan’s National Mortality Registry. The mean defined daily dose (DDD) of antipsychotics, antidepressants, mood stabilizers, and sedative-hypnotics, were calculated and survival analyses were conducted.

**Results**. A total of 102,964 individuals (54,151 men, 52.59%) with schizophrenia were included. Compared to patients with no exposure to antipsychotics, those with antipsychotic exposure had better survival outcomes, regardless of antipsychotic dosage. Antidepressant exposure, in low and moderate dosage, was associated with decreased all-cause mortality; exposure to mood stabilizers appeared to be associated with an increase in all-cause mortality. Although 89.7% of the patients had been prescribed sedative-hypnotics, exposure to sedative-hypnotics was associated with a dose-related increased mortality risk [hazard ratio (HR) in low dose group: 1.16, 95% confidence interval (CI), 1.07–1.27; HR in moderate dose: 1.32, 95% CI, 1.21–1.44; HR in high dose: 1.83, 95% CI, 1.67–2.01)].

**Conclusions**. The results indicate that in the treatment of schizophrenia, antipsychotics and antidepressants are associated with lower mortality when using adequate dosages and mood stabilizers and sedative-hypnotics with higher mortality compared with no use. Furthermore, exposure to sedative-hypnotics is associated with a dose-related increased mortality risk which warrants clinical attention and further study.

Key words: antipsychotic, antidepressant, mood stabilizer, sedative-hypnotic, daily defined dosage, mortality

**Introduction**

Compared with the general population, increased mortality has been consistently reported in individuals with schizophrenia, including deaths of natural and unnatural causes (Chang et al., 2021; Laursen, 2019; Laursen, Nordentoft & Mortensen, 2014). Patients with schizophrenia have a higher mortality risk than those suffering from other psychiatric disorders (Vermeulen et al., 2017). The life expectancy of individuals with schizophrenia is approximately 13–20 years shorter than that of the general population (Chang et al., 2021; Laursen et al., 2014; Pan, Yeh, Chan & Chang, 2020). Among causes of death in patients with schizophrenia, cardiovascular disease (CVD), cancer, and suicide predominate (Piotrowski et al., 2017). A sedentary lifestyle, smoking, obesity, adverse effects of medications, and inadequate access to health care services have also contributed to the increased mortality rates (Lahti et al., 2012; Laursen et al., 2014; Morgan et al., 2014; Pan, Yeh, Chan & Chang, 2017).

Research exploring the effects of psychotropic medications on mortality risk in schizophrenia has mainly focused on comparisons between the use and nonuse of a single category of psychotropic agent, and the follow-up duration has mostly been short. Studies have reported a decreased mortality risk in patients with schizophrenia who used antipsychotics compared with those who did not (Cullen et al., 2013; Taipale et al., 2018; Tenback, Pijl, Smeets, Os & Harten, 2012; Strømme et al., 2021). Among the few studies (Tiihonen, Mittendorfer-Rutz, Torniainen, Alexanderson & Tanskanen, 2016; Torniainen et al., 2015) that have explored the effects of the accumulative exposure dosage of psychotropic agents, a Swedish study identified a U-shaped curve in the overall mortality in relation to an increase in accumulative antipsychotic exposure (Torniainen et al., 2015). Despite that use and dosage of antipsychotics may be influenced by many factors including disease severity, no prior studies have explored the associations between accumulative antipsychotic exposure and longer-term mortality in patients with schizophrenia when taking into consideration disease severity and concomitant medications.

Polypharmacy, the simultaneous use of two or more types of psychotropic medications—for instance, some combination of an antipsychotic, antidepressant, mood stabilizer, or sedative-hypnotic—has been commonly employed in clinical practice settings, despite a lack of robust evidence concerning its efficacy (Lin, 2020; Malandain et al., 2018). Of patients who have been treated with antidepressants or mood stabilizers after their first schizophrenic episode, 35.4% started using antidepressants and 14.1% used mood stabilizers within 3 years of diagnosis (Puranen, Koponen, Tanskanen, Tiihonen & Taipale, 2020). Benzodiazepine use is also highly prevalent (79.2%) among patients with schizophrenia in Taiwan and a substantial proportion of users (62.9%) are shown to be long-term users, who may be at higher risk of drug-drug interactions with other psychotropic medications (Wu, Lin & Liu, 2011). The effects of using antidepressants, mood stabilizers, and sedative-hypotic agents on longer-term mortality of patients with schizophrenia warrant further research and clinical attention (Ballon & Stroup, 2013; Tiihonen et al., 2016).

Although an examination of the effects of one psychotropic medication on longer-term mortality that takes into account the concomitant use of other psychotropic agents is ideal, very few studies have examined these relationships in such a manner (Tiihonen et al., 2016). Therefore, the current study examined the effect of both the use and cumulative dosage of antipsychotics, antidepressants, mood stabilizers, and sedative-hypnotics on the risk of all-cause and specific-cause mortality while considering the concomitant psychotropic medications and proxies for disease severity in a national cohort of patients with schizophrenia in Taiwan.

**Methods**

Taiwan has a population of approximately 23 million people and its National Health Insurance (NHI) program is a single-payer compulsory social insurance system that centralizes the reimbursement of health care funds and guarantees equal access to health care for all citizens and legal foreign residents. In 2018, a total of 23.8 million individuals were covered under the NHI program, with a coverage rate of 100.0% (National Health Insurance Administration, 2018). The NHI maintains claims data in the National Health Insurance Research Database (NHIRD), which contains information on insured residents, including expenditures, medical procedures, medications, and basic demographic characteristics. The *International Classification of Diseases, Ninth Revision, Clinical Modification* (*ICD-9-CM*) was used to classify diagnoses in the NHIRD before 2016 (National Health Insurance Administration, 2016).

Participants aged ≥ 15 years who were diagnosed with schizophrenia (*ICD-9-CM* category 295) in 2010 were identified from Taiwan’s NHIRD (provided by the Health and Welfare Data Science Center of the Ministry of Health and Welfare in Taiwan). The identified cohort was a nationwide cohort with mixed prevalent and incident cases of schizophrenia. These identified patients with schizophrenia were followed up for 5 years, from 2010–2014. The index date was defined as the date on which a participant was first diagnosed with schizophrenia in 2010 and the observation period began on the index date. Mortality outcomes and causes of deaths were linked through Taiwan’s National Mortality Registry. We extracted data on age, sex, and socioeconomic variables, including being from a low-income household, insurance premium [monthly salary-based income of the insured, categorized into four levels: >NT$72,801, NT$36,301–72,800, NT$17,281–36,300, and <NT$17,280 (NT$=New Taiwan Dollar)], possession of a catastrophic illness certificate, and urbanization level of residence (categorized into seven levels), on the index date.

The mean defined daily dose (DDD) of a medication is the suggested average maintenance dose per day for a drug used for its main purpose in adults; the DDD was determined with reference to the guidelines of the World Health Organization (WHO Collaborating Centre for Drug Statistics Methodology, 2012). We calculated the mean DDD of antipsychotics, antidepressants, mood stabilizers, and sedative-hypnotic agents by dividing the accumulative dosages by follow-up days, and then we categorized each medication into four groups as follows: no exposure, low exposure (0–0.5 DDD), moderate exposure (0.5–1.5 DDD), and high exposure (> 1.5 DDD). Survival analyses with Cox regression were conducted to examine the effects of the use and dosage of psychotropic medications on all-cause mortality and specific-cause mortality in the cohort. Exposure variables included age, sex, socioeconomic status (insurance premium level, low-income household, and urbanization level), nonpsychiatric health care costs during the first year after diagnosis—a proxy for general medical conditions, proxies for disease severity (possession of a catastrophic illness card and psychiatric ward admission during the first year of diagnosis), and concomitant psychotropic agents usage. Statistical significance was set at P < 0.05. All statistical analyses were performed using SPSS (version 21.0; IBM, Armonk, NY, USA).

**Results**

A total of 102,964 individuals with schizophrenia were included in this study. The estimated prevalence of schizophrenia was approximately 0.45%, which is comparable to prior estimations based on Taiwan’s NHI database (Chien et al., 2004). The demographic and clinical characteristics of the cohort are summarized in Table 1. A total of 48,813 (47.4%) of the participants were women; the mean age was 44.8 years on the index date; and 12.8% of the participants were from low-income households. Among all participants, 21.5% (n = 22,176) had been admitted to the psychiatric ward within the first year of diagnosis, which serves as a proxy for disease severity. During the 5-year follow-up, 7.5% (n = 7,730) of the participants died. Of all those who died during the follow-up period, 79.9% (n = 6,176) died of natural causes: 20.2% (n = 1,248) died of CVD, 17.5% (n = 1,083) died of cancer, and 7.3% (n = 449) died from complications of diabetes mellitus (DM). Another 16.3% (n = 1,258) died of unnatural causes, including suicide (n = 798, 63.4%) and accidental death.

[Please insert Table 1 here]

The percentage of users of each psychotropic agent in each DDD group are summarized in Supplementary figure 1. Among all participants with schizophrenia, 8.5% (n = 8,733) had no exposure to antipsychotics, indicating that these patients remained untreated during the follow-up period, 32.4% (n = 33,403) had low exposure, 40.6% (n = 41,811) had moderate exposure, and 18.5% (n = 19,017) had high exposure. A total of 43,157 (42.3%) patients with schizophrenia were prescribed antidepressants. Around 29.5% (n = 30,375) of the patients used mood stabilizers; those using mood stabilizers tended to use low and moderate doses, and only 686 (0.7%) patients had high exposure. Notably, a high proportion of patients (89.7%) with schizophrenia used sedative-hypnotics during the follow-up period.

[Please insert Supplementary Figure 1 here if indicated]

The contributions of the cumulative exposure effect of each category of psychotropic agents on all-cause mortality in individuals with schizophrenia are illustrated in Table 2 and Figure 1. Compared with individuals with no exposure to antipsychotics, those with antipsychotic exposure had better survival outcomes, regardless of antipsychotic dosage. A moderate antipsychotic exposure was associated with the most hazard ratio (HR) reduction at 0.74 (95% CI=0.68–0.80). Antidepressant exposure in low and moderate doses was associated with decreased all-cause mortality in individuals with schizophrenia, and low dose exposure had the most reduction with an HR of 0.83 (95% CI=0.79–0.88). Exposure to mood stabilizers in low and moderate doses was associated with an increased all-cause mortality risk, with HRs of 1.10 (95% CI=1.04–1.16) and 1.14 (95% CI=1.03–1.27), respectively. Sedative-hypnotic exposure resulted in a significant dose-related increase curve for mortality risk, and the HR of low, moderate, and high doses were 1.16 (95% CI=1.07–1.27), 1.32 (95% CI=1.21–1.44), and 1.83 (95% CI=1.67–2.01), respectively. High exposure to sedative-hypnotics was associated with an 83% higher mortality risk compared with no exposure.

[Please insert Table 2 and Figure 1 here]

The contributions of the cumulative exposure effect of each category of psychotropic agent on mortality risk due to CVD in individuals with schizophrenia are presented in Table 2 and Figure 2. The moderate exposure group of antipsychotics had the most reduction in HR (0.80, 95% CI=0.66–0.97), which was statistically significant. Exposure to antidepressants appeared to be associated with decreased CVD mortality, with HRs of 0.72 (95% CI=0.62–0.82), 0.81 (95% CI=0.65–1.02), and 0.65 (95% CI=0.44–0.89) for low, moderate, and high doses, respectively. The high dose of antidepressant use is related to an up to 37% decreased CVD mortality. Low exposure and moderate exposure of mood stabilizers were shown to be associated with increased CVD mortality. Low-dose exposure to sedative-hypnotics appeared to be associated with decrease in the CVD mortality risk, exhibiting a statistically significant HR of 0.8; however, high dose exposure to sedative-hypnotics was associated with an increase in CVD mortality (HR 1.32, 95% CI=1.07–1.62).

[Please insert Figure 2 here]

The contributions of the cumulative exposure effect of each category of psychotropic agent on the suicide mortality in individuals with schizophrenia are presented in Table 2 and Figure 3. Antipsychotic exposure, regardless of dosage, was not related to suicide mortality. Antidepressant exposure was associated with increased suicide mortality, with an HR of 1.29 (95% CI=1.02–1.63) and 1.61 (95% CI=1.24–2.10) in the moderate- and high-dose groups, respectively, which might be partially attributable to severity of the illness that those individuals with suicidal behaviors are more likely to be prescribed antidepressants. Exposure to mood stabilizers was associated with decreased suicide in patients with low and moderate doses of mood stabilizers, and the largest reduction appeared in the moderate exposure group (HR 0.68, 95% CI=0.50–0.93). Using sedative-hypnotics, however, appears to be related to increased suicide rates in individuals with schizophrenia, with statistically significant HRs of 2.01 (95% CI=1.41–2.87) and 3.70 (95% CI=2.60–5.26) in the moderate- and high-dose groups, respectively.

[Please insert Figure 3 here]

**Discussion**

To the best of our knowledge, the current study is the first to investigate the association between longitudinal mortality risk and cumulative exposure for all major categories of psychotropic medications, namely antipsychotics, antidepressants, mood stabilizers, and sedative-hypnotics, in individuals with schizophrenia. Furthermore, this is the first observational study on the relationship between psychotropic medications and causes of death using a large national cohort from an Asian country. The results indicated that compared with individuals with no exposure to antipsychotics, those with antipsychotic exposure, regardless of dosage, had better survival outcomes in all-cause mortality. Sedative-hypnotic exposure exhibited a dose-related increase in mortality, and high exposure to sedative-hypnotics was associated with an 83% higher mortality rate compared with no exposure when controlling for use of concomitant psychotropic medications.

This research revealed that compared with schizophrenia individuals with no exposure to antipsychotics, those who were exposed to antipsychotics, regardless of dosage, had better overall survival outcomes and that patients who were exposed to a moderate dose had the largest reduction of HR at 0.74. Similarly, a previous retrospective cohort study demonstrated that antipsychotic continuity is associated with a reduced risk of mortality in schizophrenia compared with nonuse (Cullen et al., 2013). A Dutch meta-analysis revealed that individuals with schizophrenia who were exposed to antipsychotics exhibited a decreased long-term mortality risk, with a pooled risk ratio of 0.57 favoring any exposure to antipsychotics (Vermeulen et al., 2017). With regards to specific antipsychotics, not use of clozapine in treatment-resistant schizophrenia was shown to be related to a nearly two-fold mortality risk compared with those treated with clozapine (Wimberley et al., 2017). As one of the rare prior studies examining the effect of cumulative dosage of antipsychotic use in all-cause mortality in patients with schizophrenia, a Swedish national study revealed a decreased all-cause mortality risk in patients with moderate and high antipsychotic exposure (Tiihonen et al., 2016). In this regard, the present study adds to the existing literature by demonstrating that individuals with schizophrenia have decreased all-cause mortality with antipsychotic treatment, regardless of dosage, compared to those with no antipsychotic usage, based on data from real-world clinical settings in Taiwan.

Among psychotropic medications, antipsychotics have the greatest potential to adversely affect physical health (Correll, Detraux, De Lepeleire & De Hert, 2015; Taylor & Horowitz, 2020) and are shown to be associated with a dose-related increased cardiovascular risk, including sudden cardiac death and ventricular arrhythmia (Ray, Chung, Murray, Hall & Stein, 2009; Wu, Tsai & Tsai, 2015). Nevertheless, antipsychotic exposure appeared to be associated with a decreased cardiovascular mortality risk in patients with schizophrenia in our research, which was statistically significant in the moderate exposure group with an HR of 0.80. A prior Finnish report has demonstrated that users of second-generation antipsychotics and most first-generation antipsychotics did not have an increased risk of cardiovascular death compared with nonusers of antipsychotics (Kiviniemi et al., 2013). A recent Korean national cohort study has shown that individuals with schizophrenia prescribed with antipsychotics had significantly lower rates of death from certain cardiovascular illnesses than untreated patients (Oh, Nam, Park, Chae, & Kim, 2021). Consistent with our results, a Swedish national database study has revealed that exposure to antipsychotics decreased cardiovascular mortality when taken in low and moderate doses (Tiihonen et al., 2016). The aforementioned findings might result from the use of antipsychotics improving psychiatric symptoms in patients with schizophrenia, possibly leading to their enhanced ability to seek medical care and adhere to treatment and an improvement in their self-care quality and social interactions, contributing to a decreased risk of mortality, including that for CVD (Correll et al., 2015; Correll et al., 2017; Laursen et al., 2014). Given that moderate exposure to antipsychotics was related to the lowest HR in both all-cause and cardiovascular mortality in the current study, the benefits and potential risks of antipsychotics must be weighed cautiously, and prescribing antipsychotics in an adequate dosage range could be associated with decreased mortality risks in schizophrenia.

In our study, antipsychotic exposure did not contribute to suicide mortality. Review articles indicated that a lifetime risk of suicide in individuals with schizophrenia is approximately 5-10% (Hor & Taylor, 2010). Not taking any antipsychotics after the first schizophrenic episode has been shown to be associated with a 37-fold increase in suicide (Tiihonen et al., 2006). Both poor adherence to antipsychotic medication and concurrent depressive symptoms are significant risk factors for suicidal behaviors in people with schizophrenia (Sher & Kahn, 2019). However, the present study did not reveal any contribution to suicide by antipsychotic use, which may be partially attributable to the fact that the current study was based on a mixed cohort of both incident and prevalent cases of schizophrenia patients most of whom were no longer in the early phase of the disease. According to a cohort study from Taiwan, treatment with long-acting injectable antipsychotics within the first 2 years of oral antipsychotic initiation was associated with a decrease in suicide for patients with schizophrenia; however, such decrease was not observed for those who switched to long-acting injectable antipsychotics more than 2 years after initiating oral antipsychotics (Huang, Fang & Shao, 2021). Therefore, adequate antipsychotic treatment may be beneficial in reducing suicide risk, particularly for those schizophrenia patients with early phase of the disease.

Although clinical guidelines rarely recommend use of antidepressants or mood stabilizers as adjunctive treatment in individuals with schizophrenia, a recent study revealed that this population is often treated with antidepressants or mood stabilizers (Puranen et al., 2020). In the present study, individuals with schizophrenia exposed to antidepressants in low and moderate doses exhibited a significantly decreased all-cause mortality, with the lowest HR at 0.83 in those exposed to low doses. Previous studies on the effects of antidepressants on mortality risk in patients with schizophrenia have yielded mixed results. Although not reaching statistical significance, Tenback et al. reported a trend of increased all-cause mortality, with an adjusted HR of 2.11 (95% CI 0.98–4.45), based on a relatively small sample size (Tenback et al., 2012). Tiihonen et al., however, reported that any degree of antidepressant exposure was associated with lower overall mortality compared with nonuse (Tiihonen et al., 2016). Stroup et al. reported that antidepressants have positive effects in other respects, including a lower risk of psychiatric hospitalizations and emergency department visits (Stroup et al., 2019). A decreased cardiovascular mortality associated with antidepressant use in low and high dosage was found in the present study which is in line with prior studies in patients with schizophrenia (Tiihonen et al., 2016). Although data specifically on antidepressant use in patients with schizophrenia are relatively rare, evidence from research with patients with depression are informative. The excess mortality in depression is hypothesized to be associated with factors such as vascular endothelial dysfunction (Stapelberg et al., 2011), lower heart rate variability reflecting altered cardiac autonomic tone (Carney et al., 2002), and increased platelet aggregation (Stapelberg et al., 2011). Some of the aforementioned mechanisms may be also related to the adverse effects of psychotropic medications. However, antidepressant treatment may decrease overall and CVD-related mortality risks by reducing the severity and duration of depression, which can be partially attributed to the well-known association between depression and cardiometabolic health (Ditmars et al., 2021, Kemp et al., 2014, Sylvia et al., 2015). For instance, depressive symptoms are significantly associated with onset of DM, stroke, and heart disease; for individuals with these cardiometabolic diseases, depressive symptoms are further associated with increased risk of overall mortality (Qiao et al., 2021). Therefore, as revealed in the present study, in real-world clinical practice, prescribing adequate doses of antidepressants for patients with schizophrenia might be beneficial to overall and CVD mortality when it is clinically necessary.

The current study demonstrated that exposure to mood stabilizers in low and moderate doses is associated with a significant increase in all-cause mortality rates in individuals with schizophrenia, with HRs of 1.10 and 1.14, respectively. A Swedish prospective cohort study demonstrated that the concomitant use of mood stabilizers in patients with schizophrenia was associated with a higher risk of mortality (Tenback et al., 2012). A national database study in the United States also revealed that the use of a mood stabilizer after an antipsychotic medication in patients with schizophrenia was associated with an increased mortality risk with an HR of 1.31 (Stroup et al., 2019). In the present study, significantly increased CVD mortality in individuals with schizophrenia with low and moderate mood stabilizer exposure was also evident, with HRs of 1.21 and 1.46, respectively. Although speculative, mechanisms underlying the finding of increased mortality are multifactorial, which may be related to long-term adverse effects of mood stabilizers, such as weight gain, and metabolic syndrome associated with gaining weight (Gitlin, 2016; Verrotti, D'Egidio, Mohn, Coppola & Chiarelli, 2011).

A large proportion of individuals with schizophrenia (89.7%) in this study used at least one type of sedative-hypnotics during the follow-up period. Despite that current treatment guidelines recommending that benzodiazepines should not be used long-term to avoid tolerance, dependence, and dose escalation in individuals with schizophrenia (Dold, Li, Gillies & Leucht, 2013), our data suggested that long-term use of sedative-hypnotics is fairly common in Taiwan. Besides, we demonstrated that all dosages of sedative-hypnotics were associated with significantly increased all-cause mortality in individuals with schizophrenia, with the highest HR at 1.83 in patients using a high dose. The current study also found that the use of sedative-hypnotics was related to a substantial increase in suicide mortality, with a high dose being associated with the highest HR at 3.70. The combination of long half-life benzodiazepines and antipsychotics is shown to be associated with increased mortality in patients with schizophrenia (Hasan et al., 2012, Baandrup et al., 2010). Stoup et al., reported that initiating the use of a benzodiazepine after the use of a single antipsychotic was associated with an increased all-cause mortality risk (HR: 1.08) (Stroup et al., 2019). It seems also possible that when prescribing sedative-hypnotics during the treatment course of schizophrenia, their use commonly exceeds the recommended interval and becomes a regular part of the chronic treatment regimen (Peritogiannis, Manthopoulou, & Mavreas, 2016). Although a previous Dutch cohort study revealed that benzodiazepines did not affect mortality in people with schizophrenia (Tenback et al., 2012), another Swedish national cohort study, which had a larger sample size, indicated that chronic high-dose use of benzodiazepines in individuals with schizophrenia was associated with up to a 70% higher risk of death compared with patients with no exposure (Tiihonen et al., 2016). Considering the potential adverse effects of long-term use of sedative-hypnotics, other categories of medications and nonpharmacological treatment options, such as melatonin, behavior therapy and circadian rhythm adjustment, may possibly serve as adequate alternative options for patients with schizophrenia (Oliveira, Coroa & Madeira, 2019).

The strengths of the current study include a large sample size, national coverage, ability to explore numerous psychotropic agents, being the first study to include individuals with schizophrenia diagnosed in all clinical settings in an Asian country, and a longitudinal follow-up for 5 consecutive years. Several limitations should be considered when interpreting the results. First, data contained in the NHIRD only include health services provided by the NHI system in Taiwan. Some individuals with schizophrenia might have never sought medical help and were not identified in the present study. Confounding factors and selection bias due to a nonrandomized study design should be considered. Besides, the current analyses were based on earlier data, future research based on more recent dataset is warranted. The presented findings were based on data from patients with schizophrenia, the interpretations of the current results did not take into considerations any changes in the mortality curve of Taiwan’s general population over the observation period. Furthermore, diagnoses of comorbid psychiatric illnesses including substance use disorder were not assessed in this study. Because the NHIRD could not provide accurate information about the precise severity of patients’ disease, lifestyle, suicidal behaviors, and health-seeking behaviors, these factors were not assessed in this study.

In conclusion, our results demonstrated that regardless of dosage, the use of antipsychotics was associated with decreased all-cause mortality in people with schizophrenia compared with those with no antipsychotic exposure. When antidepressant exposure, in low and moderate dosage, was associated with decreased all-cause mortality; exposure to mood stabilizers in low and moderate doses appeared to be associated with a significant increase in all-cause mortality rates. Being prescribed and taking a moderate to high dosage of sedative-hypnotics was associated with higher overall mortality, including suicide mortality, in individuals with schizophrenia, which warrants clinical attention and further research to elucidate underlying causes. These results illustrate the effect of each category of psychotropic drugs on the mortality during a 5-year follow-up period in a national cohort of individuals with schizophrenia.

**Conflicts of Interest:**

The authors report no financial relationships with commercial interests.

**Acknowledgments:**

JY Lin, LL Yeh, and YJ Pan contributed to this research equally as first author. This work is based in part on data from the Health and Welfare Data Science Center, Ministry of Health and Welfare, Taiwan. The interpretation of this study does not represent the views of the Health and Welfare Data Science Center, Ministry of Health and Welfare, Taiwan.

**References**

Baandrup, L., Gasse, C., Jensen, V. D., Glenthoj, B. Y., Nordentoft, M., Lublin, H., . . . Mortensen, P. B. (2010). Antipsychotic polypharmacy and risk of death from natural causes in patients with schizophrenia: a population-based nested case-control study. *Journal of Clinical Psychiatry, 71*(2), 103-108. doi:10.4088/JCP.08m04818yel

Ballon, J., & Stroup, T. S. (2013). Polypharmacy for schizophrenia. *Current Opinion in Psychiatry*, 26, 208-213. doi: 10.1097/YCO.0b013e32835d9efb

Carney, R. M., Freedland, K. E., Miller, G. E., & Jaffe, A. S. (2002). Depression as a risk factor for cardiac mortality and morbidity: a review of potential mechanisms. *Journal of Psychosomatic Research, 53*(4), 897-902. doi:10.1016/s0022-3999(02)00311-2

Chang, C. K., Chesney, E., Teng, W. N., Hollandt, S., Pritchard, M., Shetty, H., …Patel, R. (2021). Life expectancy, mortality risks and cause of death in patients with serious mental illness in South East London: a comparison between 2008-2012 and 2013-2017. *Psychological Medicine*, 1-10. doi: 10.1017/S0033291721002257

Chien, I. C., Chou, Y. J., Lin, C. H., Bih, S. H., Chou, P., & Chang, H. J. (2004). Prevalence and incidence of schizophrenia among national health insurance enrollees in Taiwan, 1996-2001. *Psychiatry and Clinical Neurosciences, 58*(6), 611-618. doi:10.1111/j.1440-1819.2004.01311.x

Correll, C. U., Detraux, J., De Lepeleire, J., & De Hert, M. (2015). Effects of antipsychotics, antidepressants and mood stabilizers on risk for physical diseases in people with schizophrenia, depression and bipolar disorder. *World Psychiatry*, 14, 119-136. doi: 10.1002/wps.20204

Correll, C. U., Solmi, M., Veronese, N., Bortolato, B., Rosson, S., Santonastaso, P., … Stubbs, B. (2017). Prevalence, incidence and mortality from cardiovascular disease in patients with pooled and specific severe mental illness: a large-scale meta-analysis of 3,211,768 patients and 113,383,368 controls. *World Psychiatry*, 16, 163-180. doi: 10.1002/wps.20420

Cullen, B. A., McGinty, E. E., Zhang, Y., Dosreis, S. C., Steinwachs, D. M., Guallar, E., & Daumit, G. L. (2013). Guideline-concordant antipsychotic use and mortality in schizophrenia. *Schizophrenia Bulletin*, 39, 1159-1168. doi: 10.1093/schbul/sbs097

Ditmars, H. L., Logue, M. W., Toomey, R., McKenzie, R. E., Franz, C. E., Panizzon, M. S., . . . Lyons, M. J. (2021). Associations between depression and cardiometabolic health: A 27-year longitudinal study. *Psychological Medicine*, 1-11. doi:10.1017/s003329172000505x

Dold, M., Li, C., Gillies, D., & Leucht, S. (2013). Benzodiazepine augmentation of antipsychotic drugs in schizophrenia: a meta-analysis and Cochrane review of randomized controlled trials. *European Neuropsychopharmacology*, 23, 1023-1033. doi: 10.1016/j.euroneuro.2013.03.001

Gitlin, M. (2016). Lithium side effects and toxicity: prevalence and management strategies. *International Journal of Bipolar Disorders*, 4, 27. doi: 10.1186/s40345-016-0068-y

Hasan, A., Falkai, P., Wobrock, T., Lieberman, J., Glenthoj, B., Gattaz, W. F., … World Federation of Societies of Biological Psychiatry (WFSBP) Task Force on Treatment Guidelines for Schizophrenia (2012). World Federation of Societies of Biological Psychiatry (WFSBP) Guidelines for Biological Treatment of Schizophrenia, part 1: update 2012 on the acute treatment of schizophrenia and the management of treatment resistance. *The World Journal of Biological Psychiatry*, 13, 318-378. doi: 10.3109/15622975.2012.696143

Hor, K., & Taylor, M. (2010). Suicide and schizophrenia: a systematic review of rates and risk factors. *Journal of Psychopharmacology*, 24 (4 Suppl), 81-90. doi: 10.1177/1359786810385490

Huang, C. Y., Fang, S. C., & Shao, Y. J. (2021). Comparison of Long-Acting Injectable Antipsychotics With Oral Antipsychotics and Suicide and All-Cause Mortality in Patients With Newly Diagnosed Schizophrenia. *JAMA Network Open*, 4, e218810. doi: 10.1001/jamanetworkopen.2021.8810

Kemp, D. E., Sylvia, L. G., Calabrese, J. R., Nierenberg, A. A., Thase, M. E., Reilly-Harrington, N. A., . . . Iosifescu, D. V. (2014). General medical burden in bipolar disorder: findings from the LiTMUS comparative effectiveness trial. *Acta Psychiatrica Scandinavica, 129*(1), 24-34. doi:10.1111/acps.12101

Kiviniemi, M., Suvisaari, J., Koivumaa-Honkanen, H., Hakkinen, U., Isohanni, M., & Hakko, H. (2013). Antipsychotics and mortality in first-onset schizophrenia: prospective Finnish register study with 5-year follow-up. *Schizophrenia Research*, 150, 274-280. doi: 10.1016/j.schres.2013.07.043

Lahti, M., Tiihonen, J., Wildgust, H., Beary, M., Hodgson, R., Kajantie, E., … Eriksson, J. (2012). Cardiovascular morbidity, mortality and pharmacotherapy in patients with schizophrenia. *Psychological Medicine*, 42, 2275-2285. doi: 10.1017/S0033291712000396

Laursen, T. M. (2019). Causes of premature mortality in schizophrenia: a review of literature published in 2018. *Current Opinion in Psychiatry*, 32, 388-393. doi: 10.1097/YCO.0000000000000530

Laursen, T. M., Nordentoft, M., & Mortensen, P. B. (2014). Excess early mortality in schizophrenia. *Annual Review of Clinical Psychology*, 10, 425-448. doi: 10.1146/annurev-clinpsy-032813-153657

Lin, S. K. (2020). Antipsychotic Polypharmacy: A Dirty Little Secret or a Fashion? *International Journal of Neuropsychopharmacology*, 23, 125-131. doi: 10.1093/ijnp/pyz068

Malandain, L., Thibaut, F., Grimaldi-Bensouda, L., Falissard, B., Abenhaim, L., Nordon, C., & CGS study group. (2018). Correlates and predictors of antipsychotic drug polypharmacy in real-life settings: Results from a nationwide cohort study. *Schizophrenia Research*, 192, 213-218. doi: 10.1016/j.schres.2017.05.015

Morgan, V. A., McGrath, J. J., Jablensky, A., Badcock, J. C., Waterreus, A., Bush, R., … Mackinnon, A. (2014). Psychosis prevalence and physical, metabolic and cognitive co-morbidity: data from the second Australian national survey of psychosis. *Psychological Medicine*, 44, 2163-2176. doi: 10.1017/S0033291713002973

National Health Insurance Administration (2016). *Introduction of the System of the Tenth Edition of the International Classification of Diseases for National Health Insurance (ICD-10-CM/PCS)*. Retrieved 22 July 2022, from <https://www.nhi.gov.tw/Resource/webdata/27361_3_%E5%85%A8%E6%B0%91%E5%81%A5%E5%BA%B7%E4%BF%9D%E9%9A%AA%E5%9C%8B%E9%9A%9B%E7%96%BE%E7%97%85%E5%88%86%E9%A1%9E%E7%AC%AC%E5%8D%81%E7%89%88(ICD-10-CM-PCS)%E5%88%B6%E5%BA%A6%E7%B0%A1%E4%BB%8B%E5%8F%8A%E8%AA%AA%E6%98%8E.pdf>

National Health Insurance Administration (2018, December). *2018-2019 National Health Insurance Annual Report*. Retrieved 22 July 2022, from <https://www.nhi.gov.tw/resource/Webdata/2018-19%E5%85%A8%E6%B0%91%E5%81%A5%E5%BA%B7%E4%BF%9D%E9%9A%AA%E5%B9%B4%E5%A0%B1.pdf>

Oh, J., Nam, H., Park, S., Chae, J. H., & Kim, T. S. (2021). Decreased cardiovascular death in schizophrenia patients treated with antipsychotics: A Korean national cohort study. *Schizophrenia Research, 228*, 417-424. doi:10.1016/j.schres.2021.01.006

Oliveira, P., Coroa, M., & Madeira, N. (2019). Treatment Options for Insomnia in Schizophrenia: A Systematic Review. *Pharmacopsychiatry*, 52, 165-169. doi: 10.1055/a-0658-1645

Pan, Y. J., Yeh, L. L., Chan, H. Y., & Chang, C. K. (2017). Transformation of excess mortality in people with schizophrenia and bipolar disorder in Taiwan. *Psychological Medicine*, 47, 2483-2493. doi: 10.1017/S0033291717001040

Pan, Y. J., Yeh, L. L., Chan, H. Y., & Chang, C. K. (2020). Excess mortality and shortened life expectancy in people with major mental illnesses in Taiwan. *Epidemiology and Psychiatric Sciences*, 29, e156. doi: 10.1017/S2045796020000694

Peritogiannis, V., Manthopoulou, T., & Mavreas, V. (2016). Long-term Benzodiazepine Treatment in Patients with Psychotic Disorders Attending a Mental Health Service in Rural Greece. *Journal of Neurosciences in Rural Practice, 7*(Suppl 1), S26-s30. doi:10.4103/0976-3147.196447

Piotrowski, P., Gondek, T. M., Krolicka-Deregowska, A., Misiak, B., Adamowski, T., & Kiejna, A. (2017). Causes of mortality in schizophrenia: An updated review of European studies. *Psychiatria Danubina*, 29, 108-120. doi: 10.24869/psyd.2017.108

Puranen, A., Koponen, M., Tanskanen, A., Tiihonen, J., & Taipale, H. (2020). Use of antidepressants and mood stabilizers in persons with first-episode schizophrenia. *European Journal of Clinical Pharmacology*, 76, 711-718. doi: 10.1007/s00228-020-02830-2

Qiao, Y., Liu, S., Li, G., Lu, Y., Wu, Y., Ding, Y., & Ke, C. (2021). Role of depressive symptoms in cardiometabolic diseases and subsequent transitions to all-cause mortality: an application of multistate models in a prospective cohort study. *Stroke and Vascular Neurology, 6*(4), 511-518. doi:10.1136/svn-2020-000693

Ray, W. A., Chung, C. P., Murray, K. T., Hall, K., & Stein, C. M. (2009). Atypical antipsychotic drugs and the risk of sudden cardiac death. *The New England Journal of Medicine*, 360, 225-235. doi: 10.1056/NEJMoa0806994

Sher, L., & Kahn, R. S. (2019). Suicide in Schizophrenia: An Educational Overview. *Medicina (Kaunas)*, 55, 361. doi: 10.3390/medicina55070361

Stapelberg, N. J., Neumann, D. L., Shum, D. H., McConnell, H., & Hamilton-Craig, I. (2011). A topographical map of the causal network of mechanisms underlying the relationship between major depressive disorder and coronary heart disease. *Australian and New Zealand Journal of Psychiatry, 45*(5), 351-369. doi:10.3109/00048674.2011.570427

Strømme, M. F., Mellesdal, L. S., Bartz-Johannesen, C., Kroken, R. A., Krogenes, M., Mehlum, L., & Johnsen, E. (2021). Mortality and non-use of antipsychotic drugs after acute admission in schizophrenia: A prospective total-cohort study. *Schizophrenia Research, 235*, 29-35. doi:10.1016/j.schres.2021.07.009

Stroup, T. S., Gerhard, T., Crystal, S., Huang, C., Tan, Z., Wall, M., … Olfson, M. (2019). Comparative Effectiveness of Adjunctive Psychotropic Medications in Patients With Schizophrenia. *JAMA Psychiatry*, 76, 508-515. doi: 10.1001/jamapsychiatry.2018.4489

Sylvia, L. G., Shelton, R. C., Kemp, D. E., Bernstein, E. E., Friedman, E. S., Brody, B. D., . . . Calabrese, J. R. (2015). Medical burden in bipolar disorder: findings from the Clinical and Health Outcomes Initiative in Comparative Effectiveness for Bipolar Disorder study (Bipolar CHOICE). *Bipolar Disorders, 17*(2), 212-223. doi:10.1111/bdi.12243

Taipale, H., Mittendorfer-Rutz, E., Alexanderson, K., Majak, M., Mehtala, J., Hoti, F., … Tiihonen, J. (2018). Antipsychotics and mortality in a nationwide cohort of 29,823 patients with schizophrenia. *Schizophrenia Research*, 197, 274-280. doi: 10.1016/j.schres.2017.12.010

Taylor, D., & Horowitz, M. A. (2020). Antipsychotics and mortality - more clarity needed. *Psychological Medicine*, 50, 2814-2815. doi: 10.1017/S0033291720004535

Tenback, D., Pijl, B., Smeets, H., van Os, J., & van Harten, P. (2012). All-cause mortality and medication risk factors in schizophrenia: a prospective cohort study. *Journal of Clinical Psychopharmacology*, 32, 31-35. doi: 10.1097/JCP.0b013e31823f3c43

Tiihonen, J., Mittendorfer-Rutz, E., Torniainen, M., Alexanderson, K., & Tanskanen, A. (2016). Mortality and Cumulative Exposure to Antipsychotics, Antidepressants, and Benzodiazepines in Patients With Schizophrenia: An Observational Follow-Up Study. *American Journal of Psychiatry*, 173, 600-606. doi: 10.1176/appi.ajp.2015.15050618

Tiihonen, J., Wahlbeck, K., Lonnqvist, J., Klaukka, T., Ioannidis, J. P., Volavka, J., & Haukka, J. (2006). Effectiveness of antipsychotic treatments in a nationwide cohort of patients in community care after first hospitalisation due to schizophrenia and schizoaffective disorder: observational follow-up study. *BMJ*, 333, 224. doi: 10.1136/bmj.38881.382755.2F

Torniainen, M., Mittendorfer-Rutz, E., Tanskanen, A., Bjorkenstam, C., Suvisaari, J., Alexanderson, K., & Tiihonen, J. (2015). Antipsychotic treatment and mortality in schizophrenia. *Schizophrenia Bulletin*, 41, 656-663. doi: 10.1093/schbul/sbu164

Vermeulen, J., van Rooijen, G., Doedens, P., Numminen, E., van Tricht, M., & de Haan, L. (2017). Antipsychotic medication and long-term mortality risk in patients with schizophrenia; a systematic review and meta-analysis. *Psychological Medicine*, 47, 2217-2228. doi: 10.1017/S0033291717000873

Verrotti, A., D'Egidio, C., Mohn, A., Coppola, G., & Chiarelli, F. (2011). Weight gain following treatment with valproic acid: pathogenetic mechanisms and clinical implications. *Obesity Reviews*, 12, e32-e43. doi: 10.1111/j.1467-789X.2010.00800.x

WHO Collaborating Centre for Drug Statistics Methodology (2012). *Guidelines for ATC classification and DDD assignment 2013, 16th edition*. Retrieved 22 July 2022, from <https://www.whocc.no/filearchive/publications/1_2013guidelines.pdf>

Wimberley, T., MacCabe, J. H., Laursen, T. M., Sørensen, H. J., Astrup, A., Horsdal, H. T., . . . Støvring, H. (2017). Mortality and Self-Harm in Association With Clozapine in Treatment-Resistant Schizophrenia. *American Journal of Psychiatry, 174*(10), 990-998. doi:10.1176/appi.ajp.2017.16091097

Wu, C. S., Lin, Y. J., & Liu, S. K. (2011). Benzodiazepine use among patients with schizophrenia in Taiwan: a nationwide population-based survey. *Psychiatric Services*, 62, 908-914. doi: 10.1176/ps.62.8.pss6208_0908

Wu, C. S., Tsai, Y. T., & Tsai, H. J. (2015). Antipsychotic drugs and the risk of ventricular arrhythmia and/or sudden cardiac death: a nation-wide case-crossover study. *Journal of the American Heart Association*, 4, e001568. doi: 10.1161/JAHA.114.001568

**Table 1.** Demographic and clinical characteristics of patients with schizophrenia (N= 102,964) according to antipsychotic exposure

|  | Total  (n=102,964) | No Antipsychotic Exposure  (n=8,733) | Low Antipsychotic Exposure  (n=33,403) | Moderate Antipsychotic Exposure  (n=41,811) | High Antipsychotic Exposure  (n=19,017) | Significance |
| --- | --- | --- | --- | --- | --- | --- |
| Age (years old) [mean (SD)] | 44.8 (13.2) | 49.2 (13.6) | 46.6 (14.9) | 43.9 (12.0) | 41.6 (11.2) | *F*=1002.00^*^ |
| Gender [n (%)] |  |  |  |  |  | *χ*²=164.41^*^ |
| Female | 48,813 (47.4) | 3,962 (45.4) | 16,741 (50.1) | 19,554 (46.8) | 8,556 (45.0) |  |
| Male | 54,151 (52.6) | 4,771 (54.6) | 16,662 (49.9) | 22,257 (53.2) | 10,461 (55.0) |  |
| Lower-income household  [n (%)] | 13,129 (12.8) | 1,063 (12.2) | 3,706 (11.1) | 5,834 (14.0) | 2,526 (13.3) | *χ*²=144.15^*^ |
| Insurance premium level^a^  [n (%)] |  |  |  |  |  | *χ*²=476.75^*^ |
| Level (1) | 72,957 (70.9) | 5,716 (65.5) | 22,896 (68.5) | 30,465 (72.9) | 13,880 (73.0) |  |
| Level (2) | 27,137 (26.4) | 2,608 (29.9) | 9,313 (27.9) | 10,436 (25.0) | 4,780 (25.1) |  |
| Level (3) | 2,553 (2.5) | 362 (4.1) | 1,032 (3.1) | 831 (2.0) | 328 (1.7) |  |
| Level (4) | 123 (0.1) | 27 (0.3) | 47 (0.1) | 35 (0.1) | 14 (0.1) |  |
| Urbanization level^b^ [n (%)] |  |  |  |  |  | *χ*²=111.24^*^ |
| Level (1) | 25,278 (24.6) | 2,024 (23.2) | 8,306 (24.9) | 10,267 (24.6) | 4,681 (24.6) |  |
| Level (2) | 27,550 (26.8) | 2,197 (25.2) | 8,660 (25.9) | 11,352 (27.2) | 5,341 (28.1) |  |
| Level (3) | 24,727 (24.0) | 2,162 (24.8) | 8,007 (24.0) | 10,029 (24.0) | 4,529 (23.8) |  |
| Level (4) | 7,215 (7.0) | 618 (7.1) | 2,292 (6.9) | 2,898 (6.9) | 1,407 (7.4) |  |
| Level (5) | 13,323 (12.9) | 1,282 (14.7) | 4,481 (13.4) | 5,323 (12.7) | 2,237 (11.8) |  |
| Level (6) | 3,127 (3.0) | 266 (3.0) | 985 (2.9) | 1,325 (3.2) | 551 (2.9) |  |
| Level (7) | 934 (0.9) | 88 (1.0) | 344 (1.0) | 347 (0.8) | 155 (0.8) |  |
| With catastrophic illness certificate^c^ [n (%)] | 74,540 (72.4) | 5,563 (63.7) | 19,818 (59.3) | 33,285 (79.6) | 15,874 (83.5) | *χ*²=5439.55^*^ |
| Psychiatric healthcare cost [mean (SD)] | 77,661  (115,442) | 21,444  (51,271) | 40,535  (76,774) | 92,351 (120,129) | 136,530 (145,054) | *F*=4176.39^*^ |
| Non-psychiatric healthcare cost^d^ [mean (SD)] | 24,964  (91,849) | 33,458 (153,265) | 32,630 (109,254) | 19,578  (65,878) | 19,440  (64,445) | *F*=174.17^*^ |
| Psychiatric ward admission^e^ in the 1st year [n (%)] | 22,176 (21.5) | 388 (4.4) | 4,746 (14.2) | 10,523 (25.2) | 6,519 (34.3) | *χ*²=4725.24^*^ |
| Death [n (%)] |  |  |  |  |  |  |
| All causes | 7,730 (7.5) | 972 (11.1) | 3,082 (9.2) | 2,544 (6.1) | 1,132 (6.0) | *χ*²=495.38^*^ |
| Natural causes | 6,176 (6.0) | 832 (9.5) | 2,647 (7.9) | 1,945 (4.7) | 752 (4.0) | *χ*²=687.98^*^ |
| Cancer | 1,083 (1.1) | 128 (1.5) | 489 (1.5) | 335 (0.8) | 131 (0.7) |  |
| CVD | 1,248 (1.2) | 162 (1.9) | 509 (1.5) | 403 (1.0) | 174 (0.9) |  |
| DM | 449 (0.4) | 47 (0.5) | 206 (0.6) | 136 (0.3) | 60 (0.3) |  |
| Unnatural causes | 1,258 (1.2) | 120 (1.4) | 345 (1.0) | 476 (1.1) | 317 (1.7) | *χ*²=45.19^*^ |
| Suicide | 798 (0.8) | 54 (0.6) | 220 (0.7) | 314 (0.8) | 210 (1.1) |  |
| Unknown | 296 (0.3) | 20 (0.2) | 90 (0.3) | 123 (0.3) | 63 (0.3) |  |
| Follow-up days [mean (SD)] | 1729.25 (277.0) | 1678.29 (380.4) | 1713.00 (293.7) | 1744.45 (247.5) | 1747.75 (245.4) | *F*=208.36^*^ |

Continuous variables were compared using ANOVA; categorical variables were compared via chi-squared test.

^a^ Insurance premium was classified into four different levels. Level (1): Under 17,280 NTD. Level (2); Between 17,281 NTD to 36,300 NTD. Level (3); Between 36,301 NTD to 72,800 NTD; Level (4): Above 72,801 NTD.

^b^ Urbanization was classified into seven different levels. Level (1): Metropolitan City; Level (2): City; Level (3): Developing City; Level (4): Town; Level (5): Aging Population Town; Level; (6): Agriculture Town; and Level (7): Rural Area

^c^ People diagnosed by a physician as having a condition classified as a catastrophic illness by the Ministry of Health and Welfare can apply for a catastrophic certificate with which they do not need to pay a co-payment for getting care for the illness.

^d^ The non-psychiatric healthcare cost during the first year after diagnosis served as a proxy for the patient’s general physical health condition.

^e^ Admission to psychiatric ward during the first year after diagnosis served as a proxy for the severity of patient’s psychiatric illness.

SD = standard deviation; CVD = cardiovascular disease; DM = diabetes mellitus. ^*^p<0.001.

**Table 2.** Adjusted hazard ratios for antipsychotics, antidepressants, mood stabilizers, and sedative-hypnotics by DDD Group based on degree of exposure in individuals with schizophrenia

|  | **Low Exposure** | | **Moderate Exposure** | | **High Exposure** | |
| --- | --- | --- | --- | --- | --- | --- |
|  | Adjusted Hazard ratio | 95% CI | Adjusted Hazard ratio | 95% CI | Adjusted Hazard ratio | 95% CI |
| **Overall mortality** |  |  |  |  |  |  |
| Antipsychotics | 0.91* | 0.85-0.98 | 0.74** | 0.68-0.80 | 0.79** | 0.72-0.86 |
| Antidepressants | 0.83** | 0.79-0.88 | 0.91* | 0.82-0.99 | 0.98 | 0.87-1.10 |
| Mood stabilizers | 1.10* | 1.04-1.16 | 1.14* | 1.03-1.27 | 1.19 | 0.90-1.58 |
| Sedative-Hypnotics | 1.16* | 1.07-1.27 | 1.32** | 1.21-1.44 | 1.83** | 1.67-2.01 |
| **Cardiovascular mortality** |  |  |  |  |  |  |
| Antipsychotics | 0.91 | 0.76-1.09 | 0.80* | 0.66-0.97 | 0.90 | 0.72-1.14 |
| Antidepressants | 0.72** | 0.62-0.82 | 0.81 | 0.65-1.02 | 0.65* | 0.44-0.89 |
| Mood stabilizers | 1.21* | 1.05-1.39 | 1.46* | 1.13-1.88 | 1.28 | 0.60-2.70 |
| Sedative-Hypnotics | 0.80* | 0.66-0.97 | 0.99 | 0.81-1.20 | 1.32* | 1.07-1.62 |
| **Suicide mortality** |  |  |  |  |  |  |
| Antipsychotics | 1.01 | 0.74-1.36 | 0.97 | 0.72-1.31 | 1.06 | 0.77-1.45 |
| Antidepressants | 1.05 | 0.89-1.24 | 1.29* | 1.02-1.63 | 1.61** | 1.24-2.10 |
| Mood stabilizers | 0.77* | 0.65-0.91 | 0.68* | 0.50-0.93 | 1.04 | 0.57-1.91 |
| Sedative-Hypnotics | 1.21 | 0.85-1.73 | 2.01** | 1.41-2.87 | 3.70** | 2.60-5.26 |

Survival analysis was conducted by Cox regressions with controlled variables including gender, age, socioeconomic status (insurance premium level, lower-income household and urbanization level), proxy for general physical health condition (non-psychiatric healthcare cost), proxy for disease severity (catastrophic illness certificate and psychiatric ward admission during the first year), and concomitant psychotropic agents use. Hazard ratios for overall mortality, cardiovascular mortality and suicide mortality of individuals with schizophrenia were calculated by DDD group based on degree of exposure for antipsychotics, antidepressants, mood stabilizers, and sedative-hypnotics. Patients were categorized into four DDD groups: no exposure (the reference group), low exposure (<0.5DDD), moderate exposure (0.5-1.5DDD) and high exposure (>1.5DDD). CI=confidence interval. ^*^p<0.05, ^**^p<0.001.

Fig. 1. Hazard ratios and 95% confidence intervals for exposure to antipsychotics, antidepressants, mood stabilizers and sedatives-hypnotics for overall mortality^a^

^a^ Survival analysis was conducted by Cox regressions with controlled variables including gender, age, socioeconomic status (insurance premium level, low household income and urbanization level), health condition (non-psychiatric health cost), disease severity (catastrophic illness certificate, psychiatric ward admission during the first year), and concomitant psychotropic agents use. Hazard ratio for overall mortality was calculated in different degree of exposure for antipsychotics, antidepressants, mood stabilizers, and sedative-hypnotics, which were categorized into four groups with no exposure, low exposure (<0.5DDD), moderate exposure (0.5-1.5DDD) and high exposure (>1.5DDD). The scale of vertical axis was adjusted by level of hazard ratios.

Fig. 2. Hazard ratios and 95% confidence intervals for exposure to antipsychotics, antidepressants, mood stabilizers and sedatives-hypnotics for cardiovascular mortality^a^

^a^ Survival analysis was conducted by Cox regressions with controlled variables including gender, age, socioeconomic status (insurance premium level, low household income and urbanization level), health condition (non-psychiatric health cost), disease severity (catastrophic illness certificate, psychiatric ward admission during the first year), and concomitant psychotropic agents use. Hazard ratio for overall mortality was calculated in different degree of exposure for antipsychotics, antidepressants, mood stabilizers, and sedative-hypnotics, which were categorized into four groups with no exposure, low exposure (<0.5DDD), moderate exposure (0.5-1.5DDD) and high exposure (>1.5DDD). The scale of vertical axis was adjusted by level of hazard ratios.

Fig. 3. Hazard ratios and 95% confidence intervals for exposure to antipsychotics, antidepressants, mood stabilizers and sedatives-hypnotics for suicide mortality^a^

^a^ Survival analysis was conducted by Cox regressions with controlled variables including gender, age, socioeconomic status (insurance premium level, low household income and urbanization level), health condition (non-psychiatric health cost), disease severity (catastrophic illness certificate, psychiatric ward admission during the first year), and concomitant psychotropic agents use. Hazard ratio for overall mortality was calculated in different degree of exposure for antipsychotics, antidepressants, mood stabilizers, and sedative-hypnotics, which were categorized into four groups with no exposure, low exposure (<0.5DDD), moderate exposure (0.5-1.5DDD) and high exposure (>1.5DDD). The scale of vertical axis was adjusted by level of hazard ratios.

**Supplementary Fig. 1.** Percentage of users of antipsychotics, antidepressants, mood stabilizers, and sedative-hypnotics according to DDD group (Total=102,964)
