# Supplementary figures and images for: Exposure to psychotropic medications and mortality in schizophrenia: a 5-year national cohort study
Source: Psychol Med. 2022 Sep 22;53(12):5528–37. doi: 10.1017/S0033291722002732 (PMC10482725; doi:10.1017/S0033291722002732)

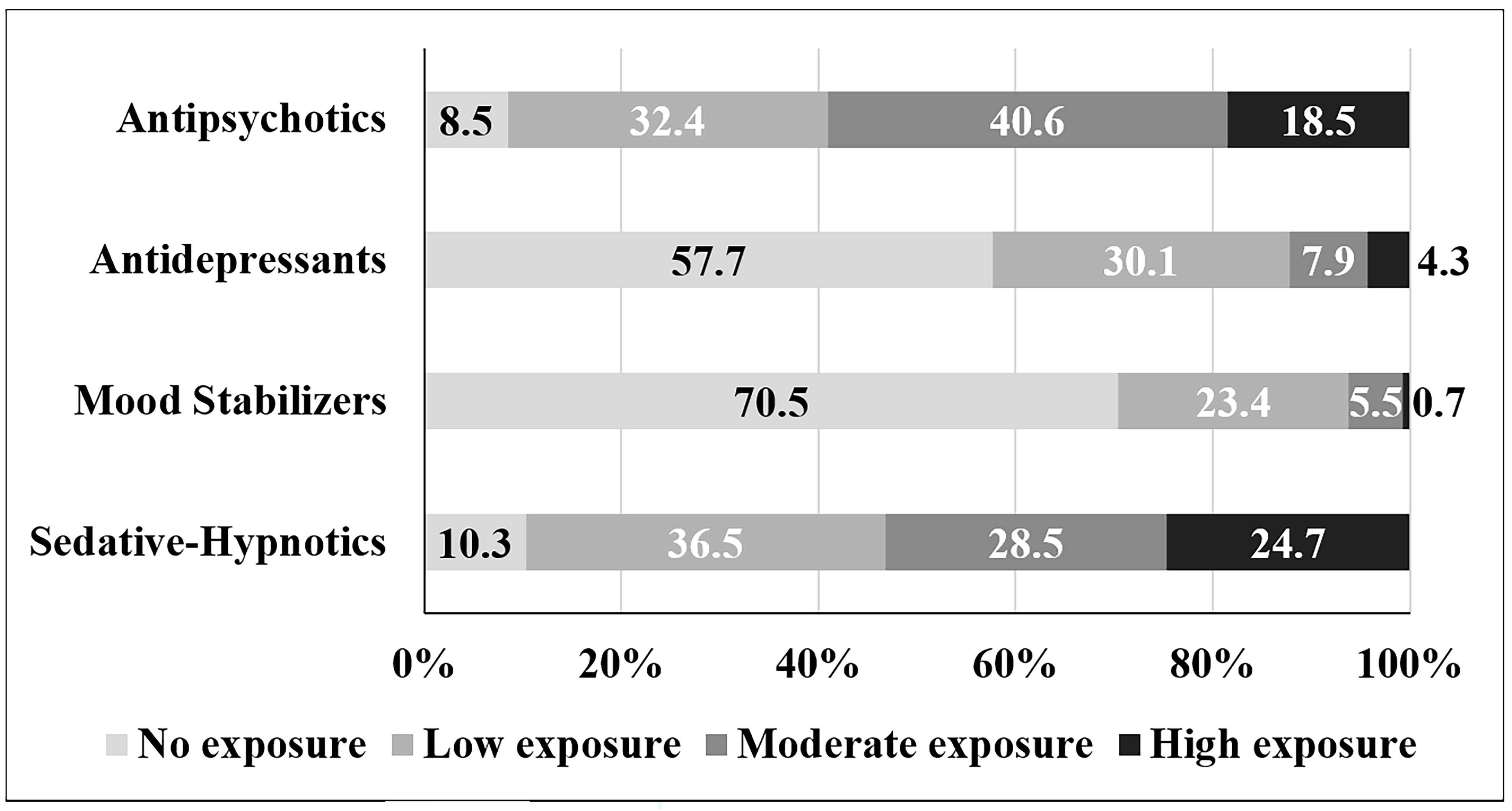

Supplement: Supplementary file 1 [file S0033291722002732sup.zip › S0033291722002732sup003.tiff]

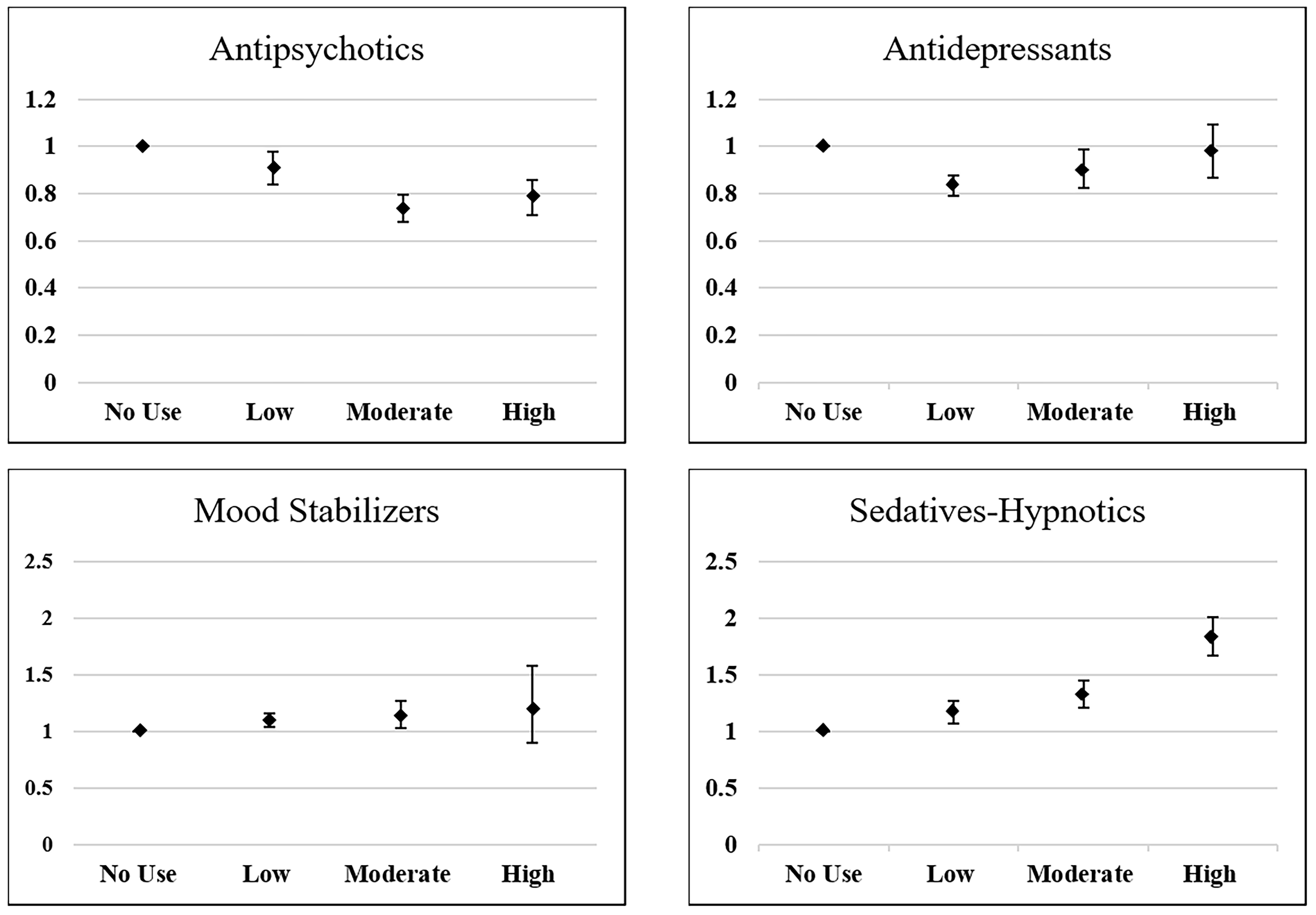

Supplement: Supplementary file 1 [file S0033291722002732sup.zip › S0033291722002732sup001.tiff]
